# Role of maintenance treatment on long-term efficacy of bilateral iTBS of the prefrontal cortex in treatment-seeking cocaine addicts: A retrospective analysis

**DOI:** 10.3389/fpsyt.2022.1013569

**Published:** 2022-11-08

**Authors:** Angela Sanna, Valentina Bini, Paola Badas, Giorgio Corona, Gabriele Sanna, Lara Marcasciano, Maria Chiara De Vivo, Marco Diana

**Affiliations:** ^1^Unitá Operativa Complessa Neurologia Riabilitativa, PO SS Trinità, ASL Cagliari, Cagliari, Italy; ^2^rTMS Italia, Cagliari, Italy; ^3^Servizio di Radiologia, Osp. Binaghi, ASL Cagliari, Cagliari, Italy; ^4^“G.Minardi' Laboratory of Cognitive Neuroscience, Department of Chemical, Physical, Mathematical and Biological Sciences, University of Sassari, Sassari, Italy

**Keywords:** cocaine use disorder, intermittent theta burst stimulation (iTBS), addiction, follow up, drop out, repetitive Transcranial Magnetic Stimulation (rTMS)

## Abstract

CUD, like other addictions, is a chronic disease characterized by a high rate of relapse and drop-out (DO) from medical and behavioral treatment programs, which is positively correlated with relapse. Repetitive transcranial Magnetic Stimulation (rTMS) protocols have shown therapeutic potential in addiction in the short term, but only a few studies have explored their long-term efficacy, so far. This study explores the long-term outcome of bilateral intermittent theta-burst stimulation (iTBS) of the prefrontal cortex (PFC) in cocaine use disorder (CUD) and the possible influence of maintenance treatment in improving abstinence and decreasing DO rates. Eighty-nine treatment-seeking CUD patients were exposed to 20 sessions of iTBS. At the end of the treatment 61 (81%) abstinent patients underwent a 12 months follow-up. Among these, 27 patients chose to follow a maintenance treatment (M), whereas 34 patients chose not to adhere to a maintenance treatment (NM). Overall, among patients reaching the 12 months follow-up endpoint, 69.7% were still abstinent and 30.3% relapsed. In NM-patients the DO rate was significantly higher than in M-ones (58.82 vs. 29.63%). The present observations show the long-term therapeutic effect of bilateral PFC iTBS to decrease cocaine consumption. Moreover, they underline the importance to perform a maintenance protocol to consolidate abstinence and decrease DO rates over time.

## Introduction

Cocaine use disorder (CUD) is a chronic, relapsing brain disease, causing health and social problems; cocaine, after cannabis, represents the second most widely used illegal substance in Europe (1, 2). As with other addictions, CUD patients show a high rate of relapse, up to 74% within the first 3 months (3, 4). Vulnerability to drug relapse depends on several factors (4, 5), among which a long-lasting reduced release of dopamine is thought to play a pivotal role (6–9). Many pharmacological and behavioral approaches have been conducted to treat CUD but the results are far from encouraging showing high dropout rates and poor medication adherence (10). Psychosocial approaches such as cognitive-behavioral therapy (CBT) and contingency management (CM) display good efficacy after short and long-term treatment in psychostimulant addiction either alone or in combination, but they are time-consuming, expensive, and often display high drop-out rates (11–14). Among the non-pharmacological strategies, repetitive Transcranial Magnetic Stimulation (rTMS) has shown therapeutic potential in treating substance and behavioral addictions targeting focal or wide bilateral areas of the brain (15). The ability of rTMS to induce long-lasting therapeutic effects relies on several mechanisms such as neurotransmitter release, modulation of synaptic activity, and expression of neurotrophic factors at the site of stimulation and in distant connected areas, thus modulating Hebbian plasticity of entire brain networks (15–17). According to the so-called “addiction cycle” described by Koob and Le Moal (18), addiction is characterized by an altered functionality of the prefrontal cortex and basal ganglia, resulting in impairment in decision making and reduced sensitivity to natural rewards; furthermore, an increase in stress-conditioned responses, modulated by the limbic system, occurs. Indeed, TMS strategies in CUD are either directed to enhance the reduced functionality of the prefrontal areas with excitatory protocols or to decrease the excessive functionality of the limbic system with inhibitory ones (19). Both approaches have shown ability in reducing cocaine intake and craving (20–24) and in modulating cue-induced responses (25), but the heterogeneity of protocols among different studies has hindered a standardization of protocols thus lowering the level of evidence for the therapeutic use of rTMS in CUD and other addictions (15). Theta-burst stimulation (TBS) protocols that mimic hippocampal endogenous theta rhythms can induce synaptic long-term potentiation (LTP) and depression (LTD), in their intermittent and continuous patterns, respectively (26, 27). TBS protocols were thought to induce longer-lasting effects on brain plasticity than conventional protocols, but a large-scale study on depression has shown a similar short- and long-term efficacy of intermittent TBS (iTBS) compared to conventional protocols in reducing depressive symptoms (28). The main advantage of this protocol is its short duration leading to more tolerability and time saving than other rTMS protocols. Indeed, iTBS has shown therapeutic potential in CUD and other forms of addiction when applied to prefrontal areas with similar efficacy as high-frequency conventional stimulation (23, 24). Nevertheless, although TBS and other rTMS protocols have shown promising results in the short term, only a few studies have studied long-term outcome and the role of maintenance treatment for consolidating long-term abstinence, with conflicting results (29–31).

Based on this evidence, the aim of this study was to retrospectively explore the long-term efficacy of bilateral prefrontal cortex (PFC) iTBS in CUD and the effect of a maintenance iTBS treatment on long-term abstinence and follow-up adherence.

## Methods

### Experimental design

**Figure 1:**
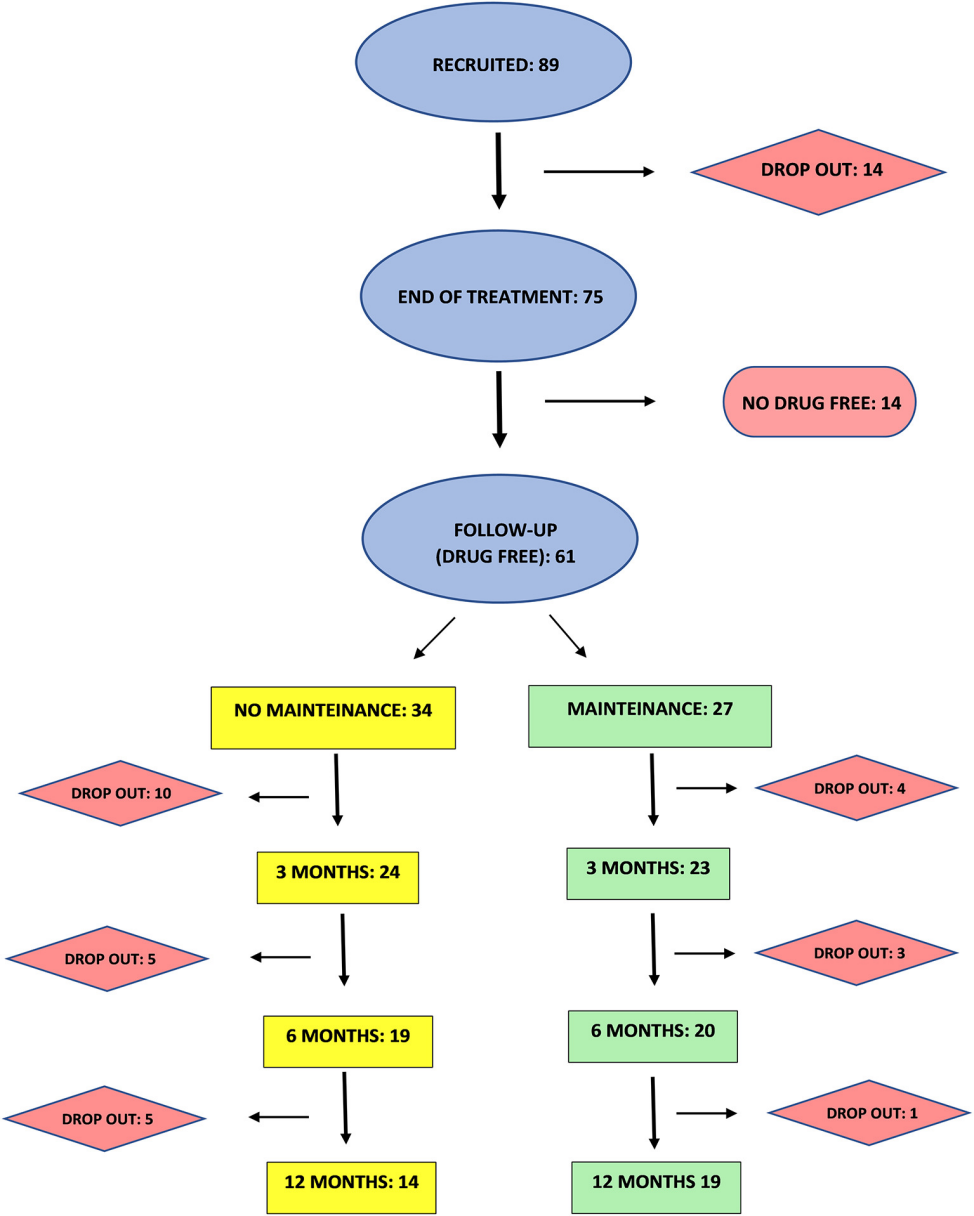
Experimental procedure flow-chart.

This is a retrospective analysis of data from clinical records of 89 CUD patients referring to an outpatient clinic from 2018 to 2021. Patients provided written informed consent to disclose their clinical data for research, anonymously. The consent form included all information regarding the nature of the TMS treatment and possible side effects. The Chief Medical Officer of the outpatient clinic approved the study and gave permission to access patients' clinical records for research scopes following Italian Legislative Decree No. 196 of June 30, 2003, “Personal Data Protection Code.” The study endorsed the Principles of Human Rights, as adopted by the World Medical Association (18th WMA General Assembly) in 1964 in Helsinki (Finland) and then amended by the 64th WMA General Assembly in 2013 in Fortaleza (Brazil). As described in Figure 1, 89 CUD patients were included in the study. Patients were treated with iTBS applied bilaterally to PFC for 20 sessions. Follow-up was performed in patients that resulted stably drug-free at the end of the treatment. Among these, 27 patients underwent maintenance sessions of iTBS (one treatment a week for 1 month followed by one treatment every 2 weeks for 2 months) while 34 drug-free patients chose not to perform a maintenance treatment. Patients and their relatives/caregivers were asked to perform urine tests at the clinic or at home once a week. Data were collected at 3, 6, and 12 months.

### Patients

Eighty-nine treatment-seeking outpatients, diagnosed according to DSM-V criteria (32) were enrolled in the study. Inclusion criteria were: age between 18 and 65 years, current CUD (i.e., have a positive urine drug screen for cocaine), motivation to stop intake, and ability to understand and sign the informed consent. Exclusion criteria were: medical devices (pacemaker, metal implants, devices for inflating), epilepsy, and pregnancy (33). The screening included medical history, physical and in-depth neurological examinations. Patients were asked about the weekly amount of cocaine consumed at baseline, at the end of rTMS treatment, and throughout follow-up; cocaine consumption was evaluated twice a week through a commercially available urine drug screen test (Home Health Ltd., Hertfordshire, United Kingdom).

### Intermittent theta burst stimulation

Magstim Rapid stimulator (Magstim Company, Whitland, Wales, UK) was used along with H4-Coil (Brainsway Ltd., Jerusalem, Israel) specifically designed to stimulate bilateral PFC and insula symmetrically (34, 35). Subjects received 20 stimulations over 4 weeks as previously described (23). ITBS protocol consisted of bursts containing 3 pulses at 50 Hz repeated at 200-ms intervals for 2 s (i.e., at 5 Hz). A 2-s train of iTBS was repeated every 10 s for 190 s and 600 pulses (26). The intensity was set at 100% of the visual resting motor threshold (RMT). For maintenance treatment 1 weekly session of iTBS was administered for 2 months.

### Statistical analysis

GraphPad Prism 8.01 software (San Diego, CA, USA) was used. To compare demographic features, multiple independent samples Student's *t*-test and Chi-Squared test were performed for normally distributed variables and categorical variables, respectively.

## Results

Table 1 shows the demographic and clinical features of patients involved in the study and the rate of side effects induced by iTBS. Treatment was well tolerated and side effects were mild and transient, when observed.

**Table 1:** Baseline socio- demographic and clinical characteristics of the sample. Data are expressed as mean and (standard deviation) or (percentage).

**Patients**		***n*** **= 89**
Gender (F/M)	F	8
	M	81
Age (yr)		36.7 (9.1)
Duration of cocaine use (years)		13.2 (7.4)
Weekly cocaine amount (g)		8.7 (7.6)
Route of administration	Inhalation	65
	Smoke	17
	Injective	7
Psychiatric comorbidities		28 (31%)
	Mood disorder	8
	Personality disorder	6
	Anxiety	14
Psychoactive prescription drugs		36 (40%)
	Mood stabilizers	9
	Benzodiazepines	11
	Antidepressants	9
	Antipsychotics	7
Other actual addictions		61 (69%)
	Nicotine	61
	Alcohol	33
	Gap	12
	Heroin	6
	Cannabis	18
rTMS side effects		28 (31%)
	Headache	10
	Dizziness	2
	Sleepiness	16
	Insomnia	9

### Effect of 20 sessions of ITBS on cocaine consumption

Eighty-nine patients were recruited, among which 14 did not complete the treatment. As shown in Figure 1, 61 (81%) of patients completing the 20 sessions treatment were found stably negative in at least three consecutive urine tests at the end of the treatment, while 14 (19 %) were still positive.

### Long-term follow-up on drug-free patients

The 61 patients resulting drug-free after 20 iTBS sessions were included in the follow-up. Segregating by maintenance treatment, as shown in Table 2, both M and NM patients display a similar rate of abstinence at 3, 6, and 12 months respectively, with no significant differences between groups for positive and negative rates at every time point.

**Table 2:** Cocaine positive and negative patients at 3, 6 and 12 months of follow up in maintenance and no-maintenance group. Data are expressed as raw data and percentages.

**Time**	**Maintenance**					**No maintenance**			
		**Negative**	**Positive**	**Drop out**	**Total**	**Negative**	**Positive**	**Drop out**	**Total**
3 months	N	17	6	4	27	18	6	10	34
	%	63.0	22.2	14.8	100.0	52.9	17.7	29.4	100.0
6 months	N	14	6	3	23	15	4	5	24
	%	60.9	26.1	13.0	100.0	62.5	16.7	20.8	100.0
12 months	N	13	6	1	20	10	4	5	19
	%	65.0	30.0	5.0	100.0	52.6	21.1	26.3	100.0

### Drop-out rates

**Figure 2:**
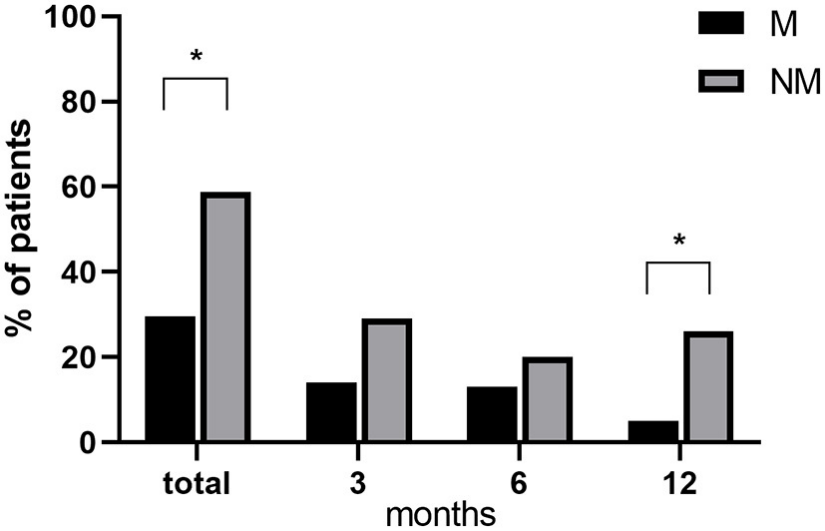
Differences in drop-out rates between M and NM at different time points. ^*^p < 0.05 M vs. NM.

Figure 2 depicts the drop-out (DO) rates at different time points in M and NM patients. A significantly higher rate of total DO in the NM group was found, reaching 58.82 % of enrolled patients vs. 29.63 % in the M group (Fisher exact test *p* = 0.04). Moreover, DO rates in the NM group show a tendency to be higher at every time point of follow-up, (as compared to the M group) reaching a statistically significant difference at 12 months (Fisher exact test *p* = 0.04; NM= 26.3%, M = 5.0 %).

## Discussion

Our data confirm our previous observation on the efficacy of bilateral iTBS in the treatment of CUD, as previously shown in a pilot study performed in a smaller sample (23). Importantly, the present data also shows that a 20-session protocol of iTBS may promote long-term abstinence in CUD patients and it suggests that a maintenance iTBS treatment increases follow-up adherence and decreases drop-out rates.

The main therapeutic issue in addiction is the long-term efficacy of pharmacological and non-pharmacological treatments; indeed, addiction is considered a chronic disease with a high relapse rate after different therapeutic approaches even in treatment-seeking patients (36–38). rTMS protocols targeting prefrontal areas have shown therapeutic efficacy in several types of addiction, including CUD, due to their ability not only in reducing drug consumption but also in ameliorating the psychological burden related to addiction (15, 22). Despite a proven short-term efficacy of rTMS treatments in CUD and other addictions, only a few studies have explored the long-term efficacy of neuromodulation: a recent metanalysis (31) shows that rTMS can reduce craving and promote abstinence from different drugs (and overeating) in short, mid and long-term. Indeed, previous studies have shown a long-term efficacy of an acute rTMS treatment in different SUDs (29, 39); on the other hand, a recent randomized, double-blind, sham-controlled multi-center study on 42 treatment-seeking CUD patients, showed no difference between real and sham stimulation in reducing cocaine craving and consumption in short and mid-term, but a marked reduction in depressive symptoms only in the real TMS patients undergoing a maintenance treatment (40). Maintenance sessions are currently proposed for different chronic disorders such as depression, but the length and frequency of sessions vary widely among different studies. Madeo et al. (30) showed that a maintenance treatment improves the efficacy of rTMS in CUD, which outlasts the reduction of treatment frequency throughout follow-up; conversely, a previous study on nicotine addiction (41) showed that rTMS can reduce cigarette smoking only in an acute setting, while the effect tends to dissipate when the sessions are less frequent.

It is well known that the heterogeneity of different studies is due to different factors, among which target area and parameters of stimulation may play an important role (8, 23, 29). Indeed, we used an iTBS protocol which is able to induce long-lasting effects on brain plasticity modulating different cellular mechanisms and whose parameters are less prone to be changed (27, 42). When considering addiction as a “*whole brain*” disease, the use of H-coil, which delivers a simultaneous stimulation of both prefrontal cortices (43), may, in theory, boost higher levels of dopamine and influence plasticity of several areas of both hemispheres which, in turn, may modulate different behavioral and cognitive processes involved in addiction neural underpinnings (8, 44). Accordingly, our data show the efficacy of bilateral iTBS of the prefrontal cortex in reducing cocaine consumption and promoting abstinence in the short and long term; moreover, they show that maintenance treatment is associated with a significantly lower percentage of drop-out rates, suggesting a better efficacy in the long term.

Drop-out is one of the main problems interfering with addiction therapies outcome (45); it has been shown that a high drop-out rate in psychosocial and pharmacological treatments is a relapse predictor (37, 45) depending on several factors, among which duration of treatment is positively associated with a better outcome (45). This is not surprising, since CUD, and SUD in general, are by definition chronic relapsing diseases and vulnerability to relapse remains high even after detoxification programs and correlates with a persistent blunted dopamine transmission and impaired executive functions (46, 47). Thus, it becomes of primary importance to keep patients' motivation and adherence to treatment to limit the relapse rate as shown in psychosocial interventions such as contingency management and cognitive-behavioral therapies (13, 48). Present data show that CUD patients undergoing a maintenance treatment display a significantly higher adherence to follow-up compared to those who did not, which display 59% of drop-out rates. Thus, based on the above-mentioned evidence, although we measured similar abstinence rates between groups, it can be inferred that the percentage of relapse to cocaine use might be higher in the NM group due to the high percentage of drop-out rates.

## Limitations

This paper has several limitations that we must recognize. First of all, this study lacks a control group since data originate from observations in treatment-seeking patients who voluntarily underwent rTMS; indeed, previous studies on SUD have shown no differences between sham and real stimulation in reducing drug intake in the short term, while significant differences emerged in the follow up (29, 49); we may speculate that is probably due to a *placebo* effect of the treatment which tend to vanish overtime. Another limit concerns the follow-up data that are mostly obtained from telephone interviews with patients and/or caregivers doing at-home urine tests. Further, in our sample the percentage of female patients is very low, which is a common problem in rTMS addiction studies (15); however, a recent paper on women with methamphetamine use disorder showed the same responsiveness to rTMS, as compared with men (50). Indeed gender differences should be better explored taking into account hormonal, psychological and cognitive indicators (51). Lastly, we did not perform any psychometric measure which might be helpful to investigate whether abstinence is accompanied by a change in mood parameters or executive functions, as already described by other authors for rTMS and other SUD treatments (5, 22, 40, 52).

## Conclusions

In conclusion, despite these limits, this paper confirms the effectiveness of iTBS in treating CUD in the short term and extends these observations to the long term. It shows that maintenance treatments may promote a better adherence to follow-up suggesting an improvement in long-term abstinence, thereby preventing relapse.

## Data availability statement

The raw data supporting the conclusions of this article will be made available by the authors, without undue reservation.

## Ethics statement

Ethical review and approval was not required for the study on human participants in accordance with the local legislation and institutional requirements. The patients/participants provided their written informed consent to participate in this study.

## Author contributions

AS, PB, GC, and MD conceived the study and designed the experiments. MD supervised the research. AS, VB, MCDV, GS, and LM performed the study. AS and VB analyzed the data. AS, PB, VB, and MD discussed the data and prepared the manuscript. All authors read and approved the final manuscript.

## Funding

This work was supported, in part, by funds FAR 2019 and FAR 2020 from Uniss to MD.

## Conflict of interest

The authors declare that the research was conducted in the absence of any commercial or financial relationships that could be construed as a potential conflict of interest.

## Publisher's note

All claims expressed in this article are solely those of the authors and do not necessarily represent those of their affiliated organizations, or those of the publisher, the editors and the reviewers. Any product that may be evaluated in this article, or claim that may be made by its manufacturer, is not guaranteed or endorsed by the publisher.
